# IgG4 disease with multiorgan involvement: a case report

**DOI:** 10.1186/s13256-024-04418-1

**Published:** 2024-02-28

**Authors:** M. N. Vidanapathirana, D. Wijayaratne

**Affiliations:** 1 Professorial Medical Unit, National Hospital of Sri Lanka, Colombo, Sri Lanka; 2 Faculty of Medicine, University of Colombo, Colombo, Sri Lanka

**Keywords:** IgG4 disease, Pulmonary, Renal, Neurological, Mononeuritis multiplex

## Abstract

**Background:**

IgG4-related disease (IgG4RD) is a rare fibroinflammatory disease with multiorgan involvement. It presents insidiously over several years and can be a diagnostic enigma. Delays in diagnosis occur due to failure to consider IgG4 as a differential diagnosis, atypical presentations, and an insidious clinical course.

**Case presentation:**

We report the case of a 70-year-old Sri Lankan man with pulmonary, renal, and neurological involvement of IgG4-related disease. Clinical manifestations evolved over a 4-year period and included exertional shortness of breath and dysesthesia of extremities. The diagnosis was established with clinical, radiological, and pathological criteria laid down by The American College of Rheumatology/European League Against Rheumatism in 2019.

Following diagnosis, the patient was started on oral steroids, with rapid improvement of his respiratory and neurological symptoms. He is currently under follow-up and will be monitored with clinical and radiological parameters, complement levels, and lung function tests.

**Conclusion:**

This case outlines the presentation of a patient with IgG4-related disease with concurrent involvement of three uncommon sites. It highlights methods of diagnostic deduction by considering the clinical course of illness, imaging, and histopathology. It also describes evolving associations of IgG4-related disease with tuberculosis and lymphomas, which bear important diagnostic and therapeutic considerations.

## Background

IgG4-related disease (IgG4RD) is a fibroinflammatory disorder with potential to involve any organ system in the body [1]. The commonly involved organs of IgG4RD are the pancreaticobiliary system, the retroperitoneal region, salivary and lacrimal glands, and lymph nodes [1].

Renal, pulmonary, and neurological involvements in IgG4RD have previously been reported, but these are not the typical sites of involvement. We report a case of IgG4RD, which ran a protracted course over 4 years with clinically evident pulmonary and neurological disease and subclinical renal involvement. Involvement of each of these organs in IgG4RD is uncommon and the involvement of all three even more unusual. We highlight the challenges of diagnosing IgG4RD in low-resource settings with high prevalence of infectious diseases, which may mimic some or all of the presentations.

## Case history

The patient was a 70-year-old Sri Lankan man with a history of hypertension and stroke. He presented to us with an unpleasant burning sensation of the distal upper and lower extremities and scalp for a duration of 3 months. There was no muscle weakness nor symptoms of autonomic neuropathy.

In his past history, he had been experiencing shortness of breath of subacute onset over the preceding 4 years and nonprogressive bilateral lower limb swelling for 6 months.

Shortness of breath had initially been episodic and exertional, and the severity had remained stable over time. He had no cough, wheeze, or identifiable precipitants. There was no fever, loss of appetite, or loss of weight. However, the shortness of breath later progressed in severity over a few months, at the time of experiencing dysesthesia.

His shortness of breath had previously been evaluated with repetitive contrast tomography, which had revealed reticulonodular pulmonary lesions with subsequent lymphadenopathy. A high erythrocyte sedimentation rate (ESR) had been detected during evaluation, and he had undergone exhaustive work-up for exclusion of tuberculosis. Details are given in Table 1.

**Table 1:** Lung-related inves﻿tigations

Investigation	Results
High-resolution computed tomography (HRCT) of the chest (T0—time of first development of dyspnea)	Reticular opacities in: -Posterior segment of right upper lobe -Superior segment of right lower lobe -Apicoposterior segment of left upper lobe Two irregular soft tissue nodules in superior segment of right lower lobe Suggestive of old healed granulomatous infection
High-resolution computed tomography (HRCT) and contrast-enhanced computed tomography (CECT) of chest (T0 + 2 years)	Multiple reticular nodular opacities in: -Posterior segment of right upper lobe -Superior segment of right lower lobe -Left superior lingular lobe Thickening of the horizontal fissure on the right side No lung masses or cavitatory lesions Pretracheal, right paratracheal, and bilateral hilar lymphadenopathy (largest: 2 cm at right hilum) Suggestive of a chronic granulomatous disease of lung ? Sarcoidosis? Tuberculosis
CECT chest and abdomen (T0 + 3 years)	Extensive bilateral diffuse centrilobular tree in bud opacities predominantly in: -Right lower segment of upper lobe -Upper segment of right lower lobe Associated with fibrotic changes and horizontal fissure thickening Diffuse ill-defined hypodense lesions without arterial phase enhancement in bilateral kidneys -Left lesion is in the mid pole: 3 cm × 4 cm × 5 cm -Right lesion is in the lower pole: 3.5 cm × 3.5 cm × 4 cm
Mantoux (T0 + 3 years)	Negative
Sputum for GeneXpert (cartridge based nucleic acid amplification test for diagnosis of tuberculosis) (T0, T0, + 2 years T0 + 3 years, T0 + 3 years)	Negative (Four times over 4 years)
BAL Gene Xpert (T0 + 3 years)	Negative
Endobronchial ultrasound (EBUS)-guided lymph node biopsy and bronchial wash (T0 + 3 years)	Reactive lymphoid proliferation
Lung function tests (T0 + 3 years)	Restrictive

*BAL* Bronchoalveolar Lavage, *CECT* Contrast Enhanced Computed Tomography, *EBUS* Endobronchial Ultrasound, *Gene Xpert* Cartridge based nucleic acid implification test for diagnosis of tuberculosis, *HRCT* High Resolution Computed Tomography

There was no contact history of tuberculosis or exposure to heavy metals or toxins. His history was not significant for symptoms suggestive of connective tissue disease or medium/small vessel vasculitis. He also did not have any risk factors for contracting viral hepatitis or human immunodeficiency virus (HIV).

On examination, his body mass index was 23 kg/m^2^. He was pale and had bilateral pitting ankle edema. There was no lymphadenopathy and no dermatological manifestations of vasculitis. His pulse rate was 80 beats per minute, with regular rhythm, and blood pressure was 140/90 mmHg without a postural drop. Cardiac auscultation was normal. Respiratory and abdominal examinations too were normal. The neurological examination revealed a right-sided hemisensory loss as a residual deficit from the previous stroke, but despite dysesthesia, there was no objective evidence of peripheral neuropathy.

His hematological and biochemical investigations during the current presentation are summarized in Table 2.

**Table 2:** Hematological and biochemical investigations

Investigation	Result	Normal range
Full blood count	Hb 8.7 (MCV 93.2, MCH 29.7) g/dL WBC 9.4 × 1000/μL Plt 398 × 1000/μL	12–16 g/dL 4–10 (× 1000/μL) 150–450 (× 1000/μL)
Peripheral blood smear	Oval and round macrocytes with normocytic normochromic cells Few hypersegmented neutrophils Platelets: normal in number and morphology Suggestive of vitamin B12/folate deficiency ± anemia of chronic disease (Levels were not done due to unavailability of reagents)	
Erythrocyte sedimentation rate	125→112→120→96 mm/hour (T0) (T0 + 2 years ) (T0 + 3 years) (T0 + 3 years )	(15 mm/hour)
Serum sodium	137 mmol/L	135–145 mmol/L
Serum potassium	4.7 mmol/L	3.5–5 mmol/L
Serum creatinine	1.1 mg/dL	0.7–1.2 mg/dL
Serum calcium	mg/dL	8.6–10.2 mg/dL
Serum magnesium	mg/dL	1.8–2.6 mg/dL
Serum phosphate	3.2 mg/dL	2.8–4.5 mg/dL
Aspartate transaminase	23 U/L	14–20 U/L
Alanine transaminase	20 U/L	29–33 U/L
Alkaline phosphatase	80 U/L	44–147 U/L
Gamma glutamyl transferase (GT)	32 U/L	0–35 U/L
Total bilirubin	mg/dL	0.1–1.2 mg/dL
Serum albumin	3.6 g/dL	3.4–5.4 g/dL
Serum globulin	3.1 g/dL	2–3.5 g/dL
Serum amylase	52.4 U/L	30–110 U/L

*Hb* Haemoglobin, *MCV* Mean Cell Volume, *MCH* Mean Cell Haemoglobin, *WBC* White Cell Count, *Plt* Platelet count

Previous labs were reviewed during the presentation, and it was found that the patient had persistently elevated ESR (> 100) over 4 years. Chest imaging had shown reticulonodular opacities in both lungs with subsequent development of paratracheal lymphadenopathy and fissure thickening. The high ESR, combined with the longstanding shortness of breath and the endemicity of tuberculosis in Sri Lanka, led to the consideration of tuberculosis as a differential diagnosis. Tuberculosis workup, including sputum for Gene Xpert, Mantoux, and paratracheal lymph node biopsy, were negative.

This patient had also previously undergone a CECT of the abdomen, which revealed bilateral renal soft tissue hypodensities, one in each kidney, following which he had undergone a renal biopsy. Upon revisiting the imaging, the renal hypodensities were discrete, round parenchymal lesions, located in the cortex without arterial phase enhancement. The differential diagnoses for such imaging findings are metastasis, renal lymphoma, infarctions, tuberculosis, and sarcoidosis. However, there was no retroperitoneal or para-aortic lymphadenopathy to have supported the first two differentials.

The renal biopsy revealed histological evidence of IgG4 disease with dense lymphoplasmocytic infiltration and storiform fibrosis resulting in tubulointerstitial nephritis (TIN). There were no granulomata noted in the histology to point toward tuberculosis and sarcoidosis. However, IgG4 staining had not been performed at this time.

During the current presentation with dysesthesia, the patient underwent nerve conduction studies and was found to have mononeuritis multiplex. Since IgG4RD became extremely likely, the renal biopsy was revisited at this time, and IgG4 staining was performed on the sample. IgG4 staining was positive with immunostaining off more than 25 cells per high power field. The cutoff for IgG4 positive cells in the kidney is 10, thus establishing a definite histological diagnosis.

Serum immunoglobulin levels were 1648 (569–1919) mg/dL. IgG4 subclasses could not be assessed due to resource constraints. C3 level was 77 (83–177) mg/dL and C4 was 9 (12–36) mg/dL.

**Fig. 1:**
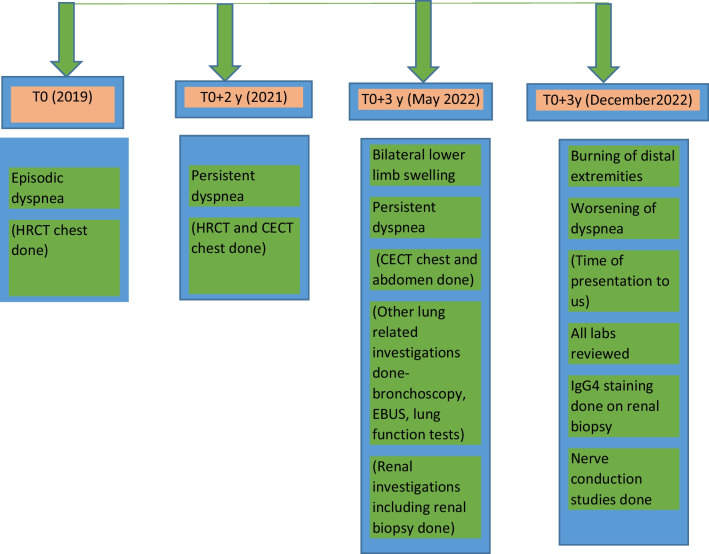
Timeline of symptoms and investigations performed

A timeline of symptom chronology with timing of investigations is shown in Fig. 1.

**Fig. 2:**
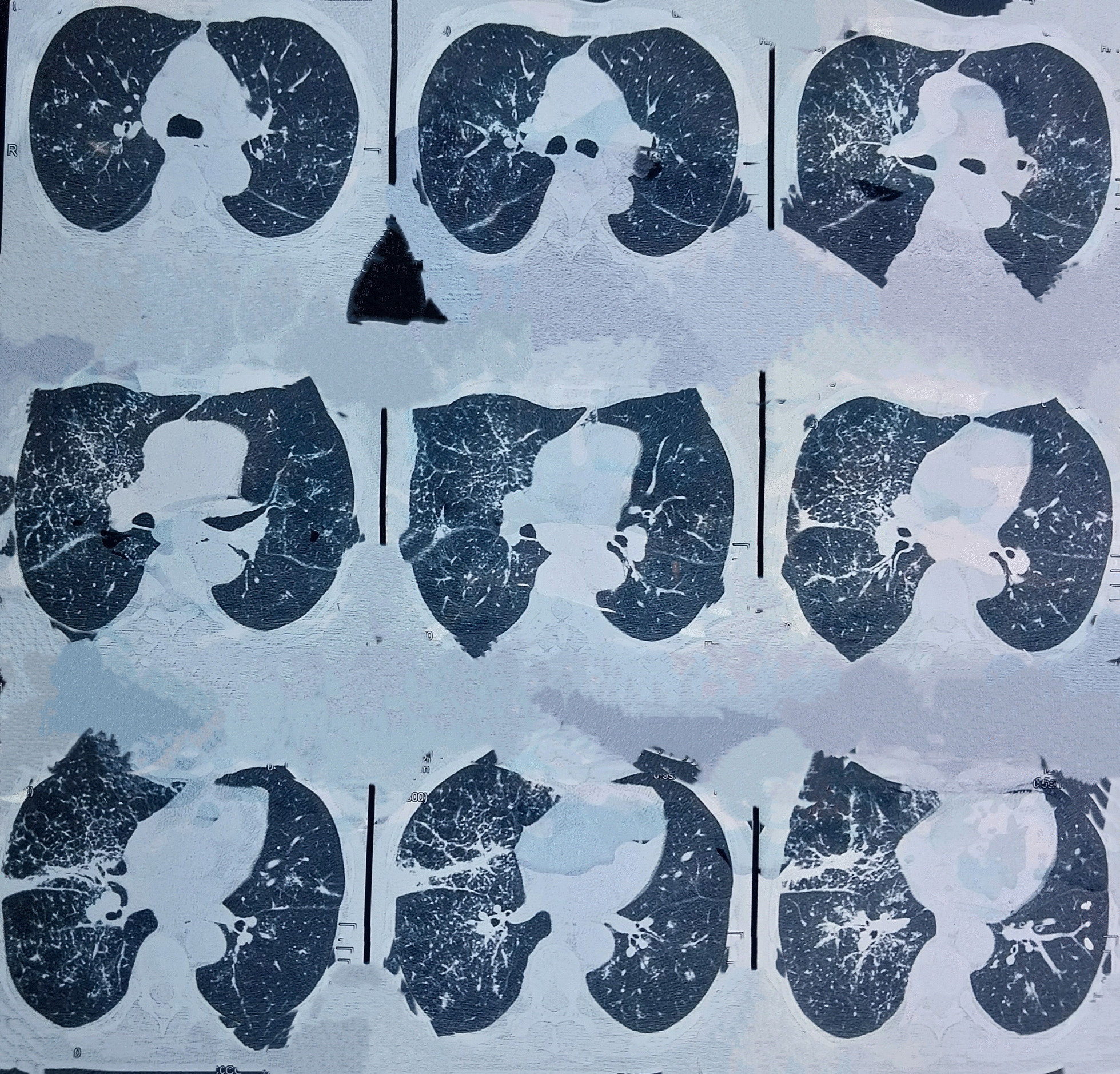
HRCT of lungs

**Fig. 3:**
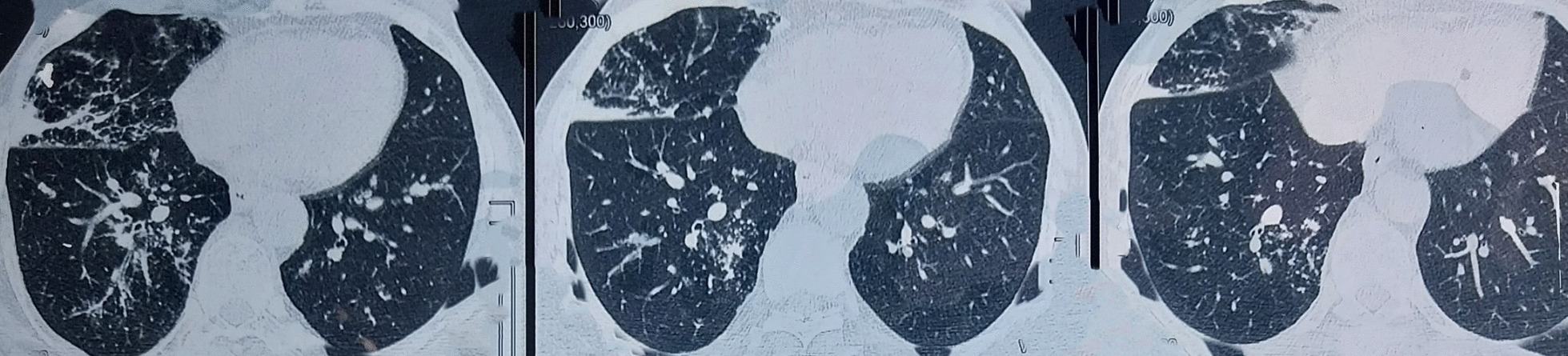
HRCT of lungs

**Fig. 4:**
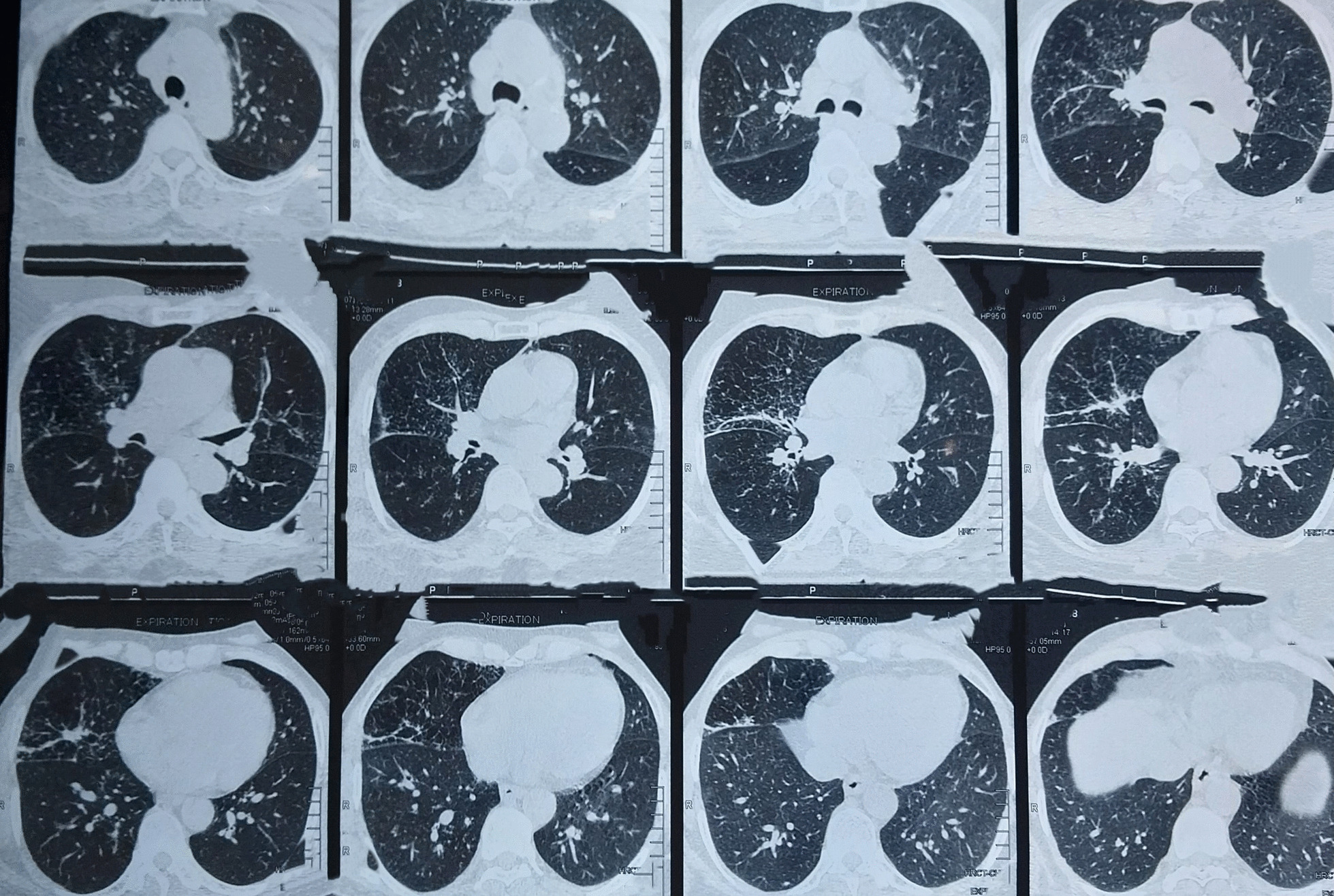
HRCT of lungs

**Fig. 5:**
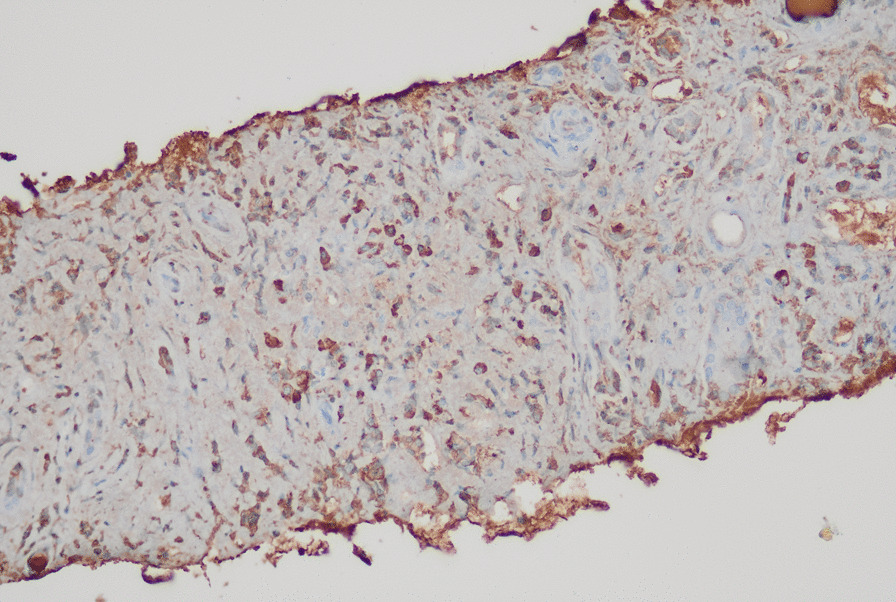
Renal biopsy with IgG4 staining

**Fig. 6:**
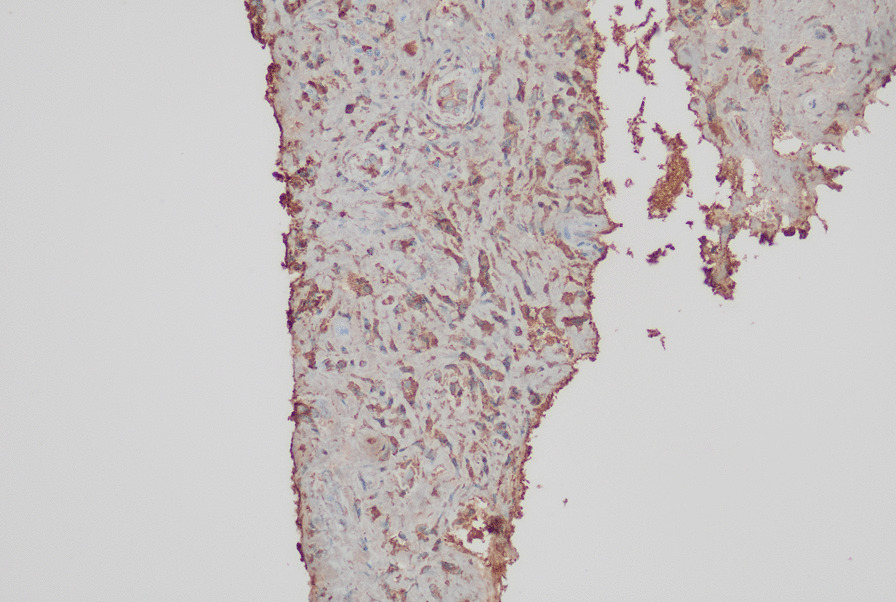
Renal biopsy with IgG4 staining

Figures 2 and 3 show the reticulonodular and fibrous changes seen in the lungs. Figure 4 shows the hypodense lesions in the kidneys. Figures 5 and 6 show the IgG4 immunostaining performed on the kidney biopsy.

The patient was diagnosed with IgG4RD based on classification criteria outlined by The American College of Rheumatology/European League Against Rheumatism in 2019 [2]. He exceeded the diagnostic cutoff value of 20 by reaching a total of 30 points. Points were obtained for histological appearance with lymphoplasmocytic infiltration, storiform fibrosis and immunostaining with IgG4 (16 points), peribronchovascular and septal thickening (4 points), and low density areas in bilateral renal cortices (10 points).

He was commenced on steroids (oral prednisolone 0.6 mg/kg/day) and showed improvement of dysesthesia and shortness of breath within the first 2 weeks. He is currently under follow-up. The criteria that will be used for assessment of progression will be clinical and radiological findings, pulmonary function tests, and complement levels.

## Discussion

IgG4RD is an immune-mediated disease that can cause fibroinflammation in nearly any organ system in the body [1]. Diagnosis is based on clinical, serological, radiological, and pathological criteria [2]. This patient met the classification criteria set forth by the American College of Rheumatology/European League Against Rheumatism in 2019 with 30 points (cutoff ≥ 20 points).

Serological criteria were not fulfilled due to unavailability of IgG4 levels. But this is neither a necessity for diagnosis nor an exclusion criterion in the absence of raised IgG4 levels, as up to 20–30% can have normal IgG4 levels [3].

The differential diagnoses that were considered during evaluation were tuberculosis, sarcoidosis, medium-small vessel vasculitis, and lymphoma.

Tuberculosis was deemed unlikely due to the indolent nature of the clinical course and negative serological, immunological, and pathological investigations. The illness ran over 4 years, with multiorgan involvement, without significant deterioration of clinical wellbeing. Such a docile picture is not in keeping with disseminated tuberculosis. IgG4RD, on the other hand, can run a benign course, with no clinical deterioration even in the presence of multiorgan involvement [4].

According to a report by Qing *et al*., tuberculosis has a link with IgG4RD, the exact nature of which has not been established yet [5]. Patients with IgG4RD were noted to have a high prevalence of tuberculosis either before the development of IgG4RD or simultaneously. This was important to consider in our patient considering the high endemicity of tuberculosis in Sri Lanka and the need to exclude it prior to initiation of high-dose steroids as treatment for IgG4RD.

Sarcoidosis and vasculitis were excluded with histology. Relevant serologies including angiotensin converting enzyme levels and antineutrophilic cytoplasmic antibody levels could not be performed due to unavailability.

With regard to the differential of lymphoma, there has been a reported association between IgG4RD and lymphomas in literature [6]. But, at present, there is no definite established etiological association between the two. Co-occurrences of IgG4RD and lymphomas have also been reported previously [7]. In this patient, the duration of the illness without significant clinical deterioration, lymphadenopathy confined to the paratracheal region without suggestive histology on biopsy safely ruled out a concurrent lymphoma in this patient.

## Pulmonary involvement in IgG4RD

The pulmonary involvement in this patient was reticulonodular opacities in bilateral lungs leading to shortness of breath. Similar radiological findings have been described in multiple reports.

Altogether, four radiological patterns for IgG4-related lung disease have been described in the report by Chen *et al*. [8]. These are mediastinal, parenchymal, pleural, and airway involvement. This patient had mediastinal involvement due to paratracheal lymphadenopathy and parenchymal involvement due to reticulonodular opacities.

The steroid response in IgG4-related lung disease is generally favorable, with respect to improvement on radiological grounds and stabilization of lung functions [9]. However, alveolar disease has the best response, with solid nodular type, which is the type in this patient, showing minimal favorable outcomes [9].

## Neurological involvement in IgG4RD

Neurological involvement in IgG4RD usually manifests in either the central or peripheral nervous system. The commonly reported manifestations are in the central nervous system with hypertrophic pachymeningitis, pituitary disease, and orbital disease [4].

The neurological involvement in this patient was of peripheral neuropathy with mononeuritis multiplex. This has been rarely reported in literature, and an extensive search revealed only two case reports [10, 11].

The rarity of reporting peripheral neuropathy may be due to a combination of rarity of existence as well as misdiagnosis as other types of vasculitis. A study by Ohyama *et al*. found that out of 149 patients who were given a diagnosis of inflammatory type of peripheral neuropathy through nerve biopsy, 29 met the required cutoff of IgG4 cell positivity necessary for diagnosis of IgG4RD. Of these 29 patients, 22 had previously been diagnosed as Anti neutrophlic cytoplasmic antibodies (ANCA)-positive vasculitis [11].

The confusion may result from the fact that peripheral neuropathy of IgG4RD also occurs by way of a vasculitic process. The vasculitis could result from either occlusion of vessels by infiltrating inflammatory cells or vasoconstriction due to significant fibrosis [10].

This patient was considered to have peripheral neuropathy due to IgG4RD in the context of overwhelming evidence for diagnosis of IgG4RD as highlighted above.

In the case report by Ohyama, the mononeuritis multiplex due to IgG4RD had excellent response to oral steroids in terms of improvement of neuropathic pain [10]. Our patient also experienced a reduction in his neuropathic symptoms within 2 weeks of commencing high dose steroids.

## Renal involvement in IgG4RD

Renal involvement in IgG4RD commonly manifests as tubulointerstitial nephritis (TIN) [1] or as renal parenchymal lesions [12]. TIN can lead to chronic renal impairment but is generally steroid responsive.

Renal parenchymal lesions appear as solitary or multiple nodules or diffuse patchy infiltrative lesions in the cortex [12]. Of these, multiple parenchymal nodules are the most common type as seen in this patient [12]. The differential diagnosis for this imaging finding is metastasis, renal lymphoma, renal infarcts, and acute pyelonephritis [12]. The first two differentials would be expected to have retroperitoneal or para-aortic lymphadenopathy, which was not seen in this patient, and acute pyelonephritis was not in keeping with this patient’s clinical picture [12]. However, renal infarcts from medium vessel vasculitis was possible, but the hypodensities found in this patient were not wedge-shaped, as would be expected in infarcts [12]. Considering that soft tissue nodules were found in both lungs and kidneys, it was decided to perform a biopsy on a renal soft tissue lesion. Ultimately, it was this that clinched the diagnosis in this patient.

This patient’s renal involvement did not manifest clinically as a decline in renal function. His renal functions and urinalysis will continue to be monitored during follow-up.

This patient had an evolving clinical course over 4 years. The involvement of three uncommon organs leading to low suspicion for IgG4RD and the indolent nature of the illness may have contributed to the diagnostic delay. Ultimately, it was the CT finding of renal soft tissue lesions and its subsequent biopsy with immunostaining that established the diagnosis. IgG4 staining was only performed on the biopsy sample later on once the patient had already developed mononeuritis multiplex. However, if the diagnosis had been suspected at the time of detecting reticulonodular lung involvement and renal parenchymal nodules, definitive diagnosis could have been pursued and steroids initiated early. This may have helped to reduce the pulmonary and neurological morbidity.

This patient was started on high-dose steroids with good clinical response. While there is little to be gained from other disease modifying therapies such as cyclophosphamide, azathioprine, and methotrexate, literature argues that there is a place for rituximab through suppression of humoral immunity, reduction of IgG4 levels, and reduction of activity of myofibroblasts, which, thereby, halts tissue fibrosis [13]. There was hesitancy for initiation of rituximab in our patient since viral hepatitis serologies were not available.

Disease recurrence depends on multiple factors including baseline IgG4 levels, number of organs involved, male sex, and tailing off steroids [13]. This patient may be at risk of recurrence considering some of these factors, and therefore, rituximab will be considered after tailing off steroids.

## Conclusion

This case is unique due to the simultaneous involvement of three uncommon sites of IgG4RD—lung, kidneys, and peripheral nerves. Consideration of IgG4RD as a differential in patients with reticulonodular lung lesions, renal parenchymal lesions, and mononeuritis multiplex will reduce disease-related morbidity due to diagnostic delays. This case also highlights important therapeutic considerations, with regard to diseases such tuberculosis and lymphoma, both of which have newly surfacing associations with IgG4RD.

## Data Availability

All data generated or analyzed during this study are included in this published article.
